# Psychosis in the Context of Travel, Substance Use, and Media Exposure: A Stress-Diathesis Perspective

**DOI:** 10.7759/cureus.107020

**Published:** 2026-04-14

**Authors:** Sowmika Boppana, Divya R Shivapuja, Alexander Perez

**Affiliations:** 1 Psychiatry, Jackson Memorial Hospital, Miami, USA

**Keywords:** alcohol use disorder (aud), brief psychotic disorder, cocaine exposure, media exposure, post-traumatic brain injury, stress-diathesis model, white matter hyperintensities

## Abstract

Brief psychotic disorder is a transient psychotic condition often precipitated by acute stress in vulnerable individuals. We describe the case of a 40-year-old woman with no prior history of psychosis who developed persecutory delusions during international travel. Her symptoms emerged in the context of multiple vulnerability factors, including heavy alcohol use, prior cocaine exposure, traumatic brain injury, and acute physiologic stress. During this period, repeated exposure to media coverage of violent crime appeared to reinforce perceived threat and escalate paranoia. Neuroimaging revealed structural abnormalities involving frontal and temporal regions, raising concern for an underlying vulnerability to psychosis. This case illustrates the stress-diathesis model of psychosis and highlights media-driven stress as a potentially underrecognized precipitating factor in susceptible individuals.

## Introduction

Brief psychotic disorder, as defined by the Diagnostic and Statistical Manual of Mental Disorders, Fifth Edition, Text Revision (DSM-5-TR), is characterized by the sudden onset of one or more psychotic symptoms lasting at least one day but less than one month, with eventual return to the individual’s premorbid level of functioning. It is an acute but transient disorder that includes one or more of the following: delusions, hallucinations, disorganized speech, grossly disorganized or catatonic behavior, and negative symptoms. Brief psychotic disorder is frequently associated with exposure to acute psychosocial stressors. Symptoms may be severe and, in some cases, associated with behavioral dysregulation or suicidal ideation. Most individuals experience complete remission with return to baseline functioning one month after the episode. The etiology of brief psychotic disorder is multifactorial, involving a complex interaction of environmental, psychological, and biological components [1]. This report presents a case of acute-onset psychosis in a 40-year-old woman with multiple vulnerability factors, including heavy alcohol use, prior cocaine exposure, traumatic brain injury, acute physiologic derangements, and prominent media-driven threat exposure during international travel. This case is notable for the role of sustained media exposure in amplifying perceived threat and contributing to the development of paranoid ideation in a vulnerable individual. We describe the clinical course, diagnostic evaluation, and management of this presentation, highlighting the complex interplay of biological and environmental factors and the importance of careful longitudinal assessment.

## Case presentation

A 40-year-old female with no prior history of psychosis presented to the Crisis Stabilization Unit (CSU) with a three-week history of paranoid delusions that began during her travels. During her trip, her behavior had been unremarkable until she sustained a head injury while participating in a polo game. She reported no loss of consciousness but did experience a headache for several days afterward. She did not undergo a formal medical evaluation and resumed her activities shortly thereafter, continuing her planned travel to the Bahamas. During her four-week stay, she engaged in extensive news consumption and frequent discussions regarding reports of increased violent crime on the island. This progressively heightened her anxiety and led to significant concerns for her safety. By the second week of her stay, she began to express fears that criminals on the island were targeting her. In the final days of her trip, her boyfriend reported an escalation of her symptoms, with the emergence of more clearly delusional beliefs. She described experiencing a burning sensation inside her lip accompanied by an abnormal taste while drinking water, which she interpreted as evidence that locals were attempting to poison her. As a result, she avoided drinking water for several days and slept only a few hours per night, believing that individuals on the island intended to harm her. She was unable or unwilling to provide specific details about these perceived threats, stating that doing so might endanger herself or us.

Fearing for her safety, the patient abruptly left the Bahamas and traveled to Miami, which she identified as a “safe space.” Despite this, her paranoia persisted. Upon noticing that the zipper of her suitcase was partially open at the airport, she became convinced that those pursuing her had connections in the United States and were continuing to track her movements. To evade surveillance, she discarded her passport and mobile phone in a trash receptacle upon leaving the airport. She then took a taxi approximately 72 miles to West Palm Beach, during which she perceived multiple police vehicles surveilling her, leading her to throw her laptop out of the moving vehicle. Still feeling unsafe, she returned to Miami in the same taxi, moving between hotels in search of affordable accommodations with the cash she had on hand. During this period, she developed a suspicion toward the taxi driver, so she abruptly exited the vehicle and left her luggage behind. She then ran more than five miles before securing a hotel room for the night. The following morning, a slight rearrangement in her room triggered renewed paranoia. The robe she hung up was now on the floor, and her shoes were in a different spot. Upon leaving the room, she became fixated on a specific guest, whom she perceived as suspicious because of his attire. She then aggressively approached the individual and forcibly removed his sunglasses, attempting to scrutinize his eyes. This behavior resulted in her being placed under an involuntary psychiatric hold (Baker Act) and admitted to an inpatient crisis unit.

During evaluation in the CSU, the patient appeared her stated age and was seated comfortably in a hospital gown. A general physical and neurologic examination was unremarkable. She was afebrile and hemodynamically stable. Cranial nerves II-XII were grossly intact, with normal motor strength, sensation, coordination, and gait. No focal neurologic deficits were identified. She exhibited no clinical signs of alcohol withdrawal, and Clinical Institute Withdrawal Assessment Alcohol Scale Revised (CIWA-Ar) scores remained low. She was calm, cooperative, and willing to answer questions. She was fully oriented and demonstrated intact attention. However, her thought process was illogical and circumstantial, and she was guarded and defensive, with prominent persecutory ideation when discussing the group she believed was targeting her. Insight was impaired, as she did not recognize her beliefs as pathological and declined treatment. Judgment was also impaired, as evidenced by her engagement in high-risk behaviors in response to perceived threats. She did not endorse suicidal or homicidal ideation. The patient declined pharmacologic treatment and expressed a desire to return to her home country. Laboratory evaluation on admission revealed multiple abnormalities (Table 1).

**Table 1:** Pertinent laboratory findings. Laboratory values on admission (04/03/2024) demonstrating elevated CPK, liver enzymes (AST, ALT, GGT), hemoglobin, hematocrit, and albumin. Elevated hemoglobin, hematocrit, and albumin are consistent with hemoconcentration in the setting of prolonged dehydration, as the patient reported no oral fluid intake since 03/29/2024. Elevated CPK suggests muscle injury, while elevated AST, ALT, and GGT may reflect hepatic stress in the setting of chronic alcohol use.

	Patient’s laboratory values (4/3/24 11:41)	Reference range (females)
General chemistry		
Creatine phosphokinase (CPK)	2,937 µg/L	10–120 µg/L
Total protein	8.9 g/dL	6.0–7.8 g/dL
Albumin	5.6 g/dL	3.5–5.5 g/dL
Total bilirubin	1.6 mg/dL	0.1–1.0 mg/dL
Liver function tests		
Aspartate aminotransferase (AST)	118 U/L	12–38 U/L
Alanine aminotransferase (ALT)	56 U/L	10–40 U/L
Gamma-glutamyl transferase (GGT)	45 U/L	5–25 U/L
Lipid profile		
High-density lipoprotein (HDL)	96 mg/dL	40–60 mg/dL
Cholesterol	245 mg/dL	Normal: <200 mg/dL; high: >240 mg/dL
Hematology		
Red blood cell (RBC) count	5.16 million/mm³	3.92–5.13 million/mm³
Hemoglobin	15.8 g/dL	12.0–15.5 g/dL
Hematocrit	46.8%	36–44%
Toxicology screen		
Acetaminophen	<10.0	-
Ethanol	<10.0	-
Cocaine and metabolites	Not detected	-
Benzodiazepines	Presumptive	-
Opiate	Not detected	-
Amphetamine	Not detected	-
Urine barbiturate	Not detected	-
Urine methadone	Not detected	-
Salicylate	<1.0	-

**Figure 1:**
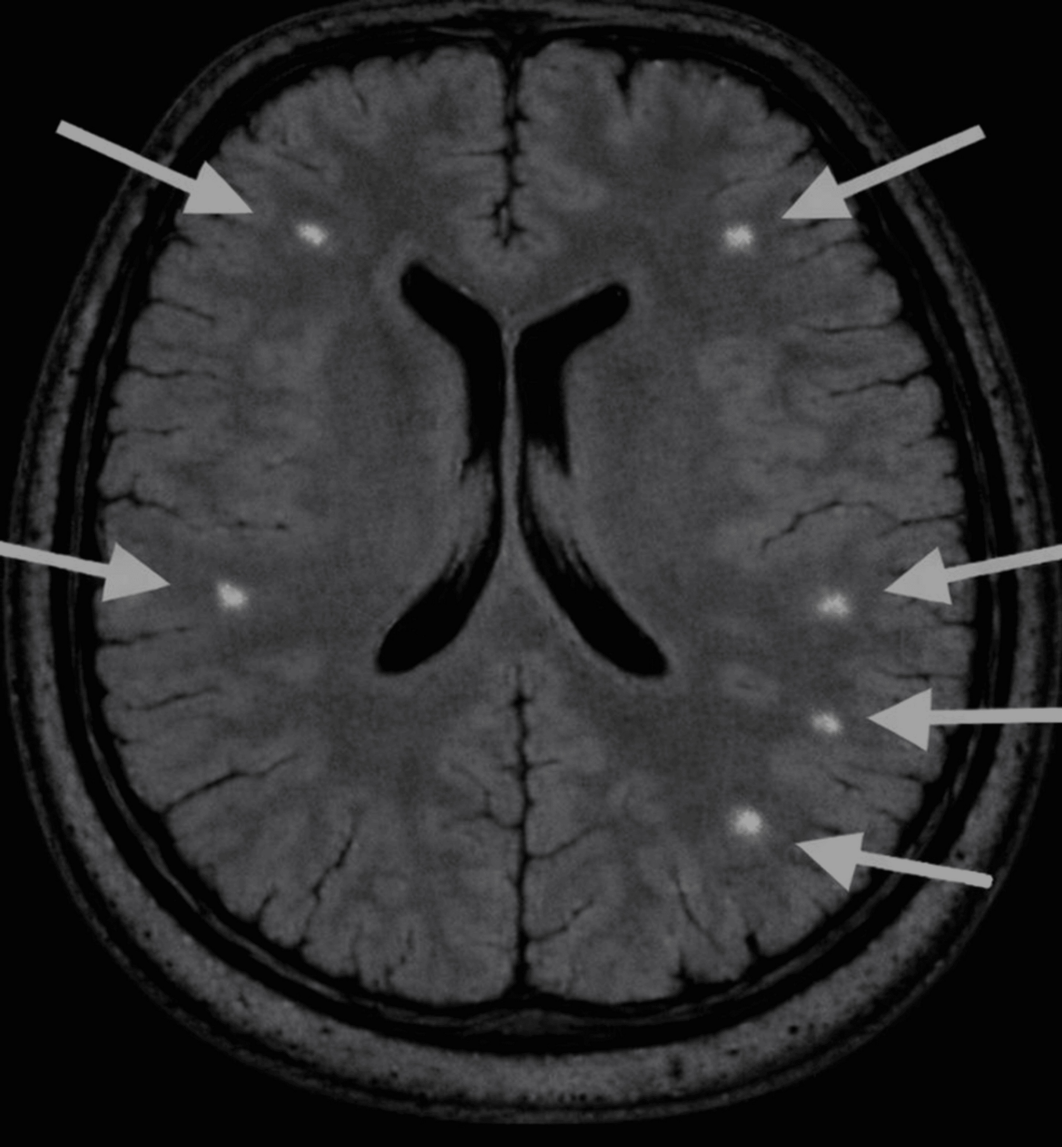
Axial brain MRI FLAIR imaging. Axial magnetic resonance imaging (MRI) of the brain using a fluid-attenuated inversion recovery (FLAIR) sequence demonstrates scattered deep white matter hyperintensities in the bilateral frontal and parietal regions, as indicated by the arrows. These findings are nonspecific and may reflect chronic small vessel ischemic changes, a demyelinating process, or inflammatory or vasculitic etiologies.

Neuroimaging was performed to evaluate possible neurological contributions to her acute psychiatric presentation. Axial magnetic resonance imaging (MRI) using a fluid-attenuated inversion recovery (FLAIR) sequence demonstrated scattered deep white matter hyperintensities in the bilateral frontal and parietal regions (Figure 1).

Before this presentation, the patient had no documented psychiatric diagnoses or medical comorbidities, aside from substance use. The patient reported daily consumption of around 750 mL of wine or champagne over the past five years. She also disclosed a history of cocaine use, using approximately 2 grams of cocaine every two to three days between the ages of 15 and 18, with a brief recurrence at age 26. Additionally, there was a family history of psychiatric illness, as her paternal aunt was diagnosed with schizophrenia, and her father had a gambling disorder.

**Figure 2:**
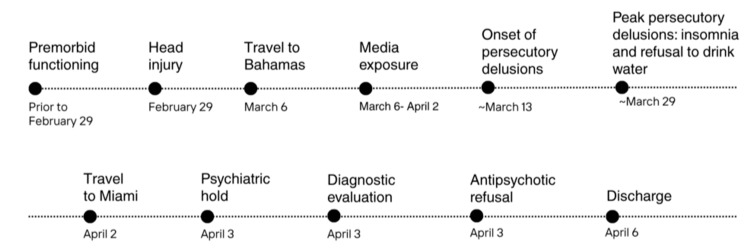
Clinical timeline of symptom onset, contributing factors, and hospital course. The figure outlines key events, including premorbid functioning, head injury, travel to the Bahamas, period of sustained media exposure, onset and peak of paranoid delusions, inpatient psychiatric hold, diagnostic evaluation, treatment refusal, and discharge.

After a 72-hour involuntary psychiatric hold (Florida Baker Act), the patient was ultimately discharged from the CSU after her mother traveled to accompany her back to their home country. At discharge, she continued to endorse persecutory, paranoid, and grandiose delusions, as well as disorganized thoughts. The patient and her family were provided with laboratory results, neuroimaging reports, and comprehensive discharge documentation, along with strong recommendations to establish follow-up with both neurology and psychiatry within one week of discharge. Figure 2 presents a clinical timeline of the case.

## Discussion

The development of brief psychotic disorder is multifactorial and may be explained by the diathesis-stress hypothesis. This theory suggests that biological and genetic predisposition, combined with environmental influences, can contribute to the development of psychosis [2]. In this case, we illustrate how the interplay of substance use, environmental factors, and traumatic brain injury may have contributed to the development of acute psychosis.

Substance use is a well-recognized trigger for psychosis [3]. The patient had a history of heavy alcohol use and prior cocaine use, both of which may have contributed to her underlying vulnerability. According to the Alcohol Abuse and Alcoholism Screening Guide, hazardous drinking is defined as more than 14 standard drinks per week or more than four drinks per occasion [4]. The patient reported a daily consumption of approximately 750 mL, equivalent to about five standard drinks per day, of wine or champagne for five years, consistent with hazardous drinking. Chronic alcohol use has been associated with dopaminergic dysregulation, impaired stress tolerance, and increased risk of psychotic symptoms, even in the absence of acute intoxication [5,6]. Despite undetectable serum ethanol on admission, long-standing heavy alcohol use may have lowered the patient’s threshold for psychotic decompensation. Although urine toxicology was positive for benzodiazepines, this finding reflected iatrogenic pharmacologic management during the patient’s involuntary psychiatric hold. The patient received a “B-52” intramuscular injection for acute agitation, consisting of haloperidol, lorazepam, and diphenhydramine. There was no evidence of chronic benzodiazepine use, and benzodiazepine exposure was therefore unlikely to have contributed to the patient’s psychotic presentation. Regarding cocaine use, the patient reported no recent use, and toxicology screening was negative; however, rare cases of persistent psychotic symptoms following cocaine exposure have been reported [7]. Taken together, substance use in this case likely functioned as a vulnerability factor within the stress-diathesis model rather than as an acute precipitant. The patient’s acute physiological stress also likely contributed to psychotic symptoms. Several days of poor oral intake resulted in dehydration with hemoconcentration, evidenced by elevated red blood cell count, hemoglobin, and hematocrit, reflecting reduced plasma volume [8]. This stress was compounded by possible alcohol-associated hepatocellular injury, suggested by aspartate aminotransferase predominance over alanine aminotransferase, which may be seen in alcohol-associated liver injury, as well as mild hyperbilirubinemia, and significant muscle breakdown with markedly elevated creatine phosphokinase [9]. In an individual with an underlying vulnerability to psychosis, these combined metabolic and systemic stressors may have lowered the threshold for psychotic symptoms [10].

The patient’s imaging revealed white matter hyperintensities in the bilateral frontal and parietal lobes on an axial (FLAIR) MRI. These findings are nonspecific, and the radiologic differential included chronic small-vessel ischemic changes, demyelinating, inflammatory, or vasculitic processes. Given the patient’s history of cocaine use, a cerebrovascular etiology was considered, as cocaine is a potent vasoconstrictor and a well-established risk factor for ischemic stroke and white matter injury [11]. In addition, the patient’s history of traumatic brain injury represents another potential contributor, as it has been associated with cerebral microvascular disruption, impaired cerebral perfusion, and secondary ischemic injury, which may manifest as focal infarcts or diffuse white matter changes on neuroimaging [12]. This structural contribution to psychosis was also considered, as cerebrovascular lesions involving frontal and temporal regions and associated white matter tracts, as seen in this patient, have been linked to post-stroke psychosis [13].

Traumatic brain injury presents with a range of neuropsychiatric symptoms, with psychosis being a rare but recognized manifestation [14]. The patient reported a significant impact to her head during a polo match, which preceded the onset of her paranoid thoughts. One study suggested that post-traumatic brain injury psychosis may result from damage to the frontal and temporal lobes upon impact [14], regions in which this patient demonstrated imaging abnormalities. Ultimately, traumatic brain injury may interact with underlying genetic vulnerabilities, contributing to the development of psychotic symptoms [14].

We theorize that media and social influence may have acted as a reinforcing psychological stressor in this case. Digital media has fundamentally transformed the landscape of human interaction, and there is growing evidence to suggest that excessive engagement with these platforms can impact mood and contribute to psychological distress [15]. On January 26th, 2024, the United States issued a level 2 travel advisory due to an increase in violent crimes, such as burglaries, armed robberies, and sexual assaults, toward tourists [16]. The patient reported engaging with news and social media coverage of these events for approximately six to eight hours per day, frequently supplemented by ongoing discussions with other expatriates on the island. She was also in close geographic proximity to the reported crimes, which may have amplified perceived threat and paranoia in a vulnerable individual within a diathesis-stress framework. Similar patterns have been described in prior case studies, in which intensive media exposure during traumatic events, such as the September 11 terrorist attacks and the Challenger space shuttle explosion, was associated with heightened psychological distress among viewers, supporting the role of media-driven stressors in contributing to psychological distress in this patient [17]. However, exposure was based on patient report, and causality cannot be established; alternative contributors, including substance use, recent head injury, and genetic predisposition, should be considered.

Differential diagnosis

Substance-induced psychosis was considered, given the patient’s history of heavy alcohol use and prior cocaine exposure. However, there was no evidence of recent cocaine use, toxicology screening was negative, and serum ethanol was undetectable on admission. The patient reported last alcohol use the day before presentation, and there were no documented signs of alcohol withdrawal. Benzodiazepine exposure was attributed to iatrogenic administration during acute management. Longitudinal assessment of symptom resolution with abstinence would be informative.

Psychosis due to another medical condition was also considered in the context of a recent head injury, post-injury headache, white matter hyperintensities on MRI, and metabolic derangements, including dehydration, elevated creatine kinase, and liver enzyme abnormalities. However, the absence of focal neurologic deficits and the lack of a comprehensive neurologic workup limit definitive attribution. Further outpatient neurologic evaluation, including electroencephalography or inflammatory studies if clinically indicated, would be appropriate. However, these were not performed due to the patient’s preference for early discharge and intent to return to her home country for ongoing care due to concerns about healthcare costs.

Delusional disorder, persecutory type, was considered, given the presence of prominent persecutory beliefs and initial relative preservation of orientation and behavior. However, the presence of disorganized thought processes and bizarre behaviors, along with the acute onset, is less consistent with this diagnosis. Longitudinal assessment of symptom organization and functional status is needed.

An emerging primary psychotic disorder, such as schizophreniform disorder, remains a consideration given the family history of schizophrenia, persistence of delusions at discharge, and disorganized thought processes. However, the acute onset in the context of multiple stressors and the current duration of symptoms of less than one month limit diagnostic certainty. Continued follow-up is essential, as persistence of symptoms beyond one month would warrant reconsideration of this diagnosis.

Management

Repeated exposure to distressing media content has been associated with increased psychological distress and symptom exacerbation in vulnerable individuals [18]. Therefore, counseling patients to limit media consumption, particularly regarding timing and frequency, may be beneficial. It may also be important to encourage patients to establish boundaries when discussing distressing sociopolitical events with family and friends.

The patient declined antipsychotic treatment. Decision-making capacity was assessed using the four-abilities model, demonstrating the ability to communicate a choice, understand relevant information, and reason about treatment options, although appreciation was partially impaired by persecutory delusions. Overall, she was determined to have sufficient capacity to refuse treatment. Risk assessment revealed no suicidal or homicidal ideation and no evidence of imminent risk during admission. Risk mitigation included involvement of her mother in discharge planning, development of a safety plan focused on recognition of worsening symptoms and help-seeking, counseling on substance avoidance, and provision of crisis resources, along with clear instructions to seek urgent psychiatric evaluation if symptoms worsen after returning to her home country. Although delusions persisted, she did not meet the criteria for continued involuntary hospitalization, as she was not an imminent danger to herself or others and was able to participate in discharge planning; therefore, discharge was deemed appropriate with plans for psychiatric and neurologic follow-up within one week.

Limitations

This case report has several limitations. First, the multifactorial nature of the patient’s presentation limits the ability to establish a definitive cause-and-effect relationship or identify a single precipitating factor. Second, although neuroimaging revealed structural abnormalities, further neurologic evaluation was not completed, limiting the interpretation of their clinical significance. Additionally, the lack of longitudinal follow-up is a significant limitation, as the patient returned to her home country shortly after discharge, preventing assessment of symptom resolution, treatment adherence, and progression of her condition. Notably, the patient was discharged while still actively delusional, and therefore did not meet the DSM-5-TR requirement for full remission within one month for a diagnosis of brief psychotic disorder. As such, the diagnosis is best considered provisional, consistent with acute psychosis of unclear etiology, with differential considerations including substance-induced or medically related psychosis. Persistence of symptoms beyond one month would warrant reconsideration of the diagnosis as schizophreniform disorder per DSM-5-TR. Longitudinal reassessment and further evaluation are necessary to clarify the diagnosis and exclude alternative etiologies.

## Conclusions

This case highlights the multifactorial nature of acute psychosis, underscoring the importance of a comprehensive approach to assessment that integrates biological vulnerability, psychosocial stressors, and media exposures. In this patient, a convergence of underlying risk factors and acute stressors appeared to lower the threshold for psychotic decompensation, consistent with the stress-diathesis model. Notably, this case highlights the potential role of media-driven stress as a precipitating factor for vulnerable individuals. Awareness of excessive exposure to distressing media content and patient-centered counseling on media consumption may represent a practical and underrecognized strategy to reduce symptom exacerbation. Further research is needed to better define the relationship between media exposure and psychosis and to inform targeted preventive interventions.
